# Supplementary material for: Effects of Storage Temperature on Indica-Japonica Hybrid Rice Metabolites, Analyzed Using Liquid Chromatography and Mass Spectrometry
Source: Int J Mol Sci. 2022 Jul 4;23(13):7421. doi: 10.3390/ijms23137421 (PMC9266784; doi:10.3390/ijms23137421)
Supplement: Supplementary file 1 [file ijms-23-07421-s001.zip › ijms-1738274-supplementary.pdf]

Supplementary Files

Effects of storage temperature on indica-japonica hybrid rice metabolites, analyzed using liquid chromatography and mass spectrometry

Lin Zhu[a†], Yu Tian[b†], Ling Jiangang[a], Xue Gong[b], Jing Sun[b*] and Li-Tao Tong[b*]

[a]Institute of Agricultural Products Processing, Key Laboratory of Preservation engineering of Agricultural Products, Ningbo Academy of Agricultural Sciences, Ningbo, Zhejiang 315040, China

[b]Institute of Food Science and Technology, Chinese Academy of Agricultural Sciences/Key Laboratory of Agro-Products Processing Ministry of Agriculture, Beijing, 100193, China

[†]Co first author

* Correspondence: author. Tel/Fax: +86-10-6281-7417 E-mail address: tonglitao@caas.cn (L. T. Tong); Tel/Fax: +86-10-6281-0295 E-mail address: ycsunjing2008@126.com (J. Sun)

No.	Content
**Table S1**	Compound names, retention times, exact mass, and KEGG IDs of detected compounds in UPLC-Qtrap-MS.
**Figure S1**	Overlaid total ion current chromatograms (TICs) of all QC samples.
**Figure S2**	Overlaid extracted ion current chromatograms (EICs) of internal standard 2-chlorophenylalanine in QC samples.

Table S1. Compound names, retention times, exact mass, and KEGG IDs of detected compounds in UPLC-Qtrap-MS.

No.	compound name	Rt(min)	KEGG_ID	EXACT_MASS
1	5,7-Dihydroxyisoflavone	14.24	C02168	241.1103
2	8-Azabicyclo-3.2.1-octan-3-ol	14.09		127.0997
3	Astrocasine	13.94	C10130	310.2045
4	5-Tricosyl-1,3-benzenediol	13.85		432.3967
5	Oxoglaucine	13.83		351.1107
6	Venlafaxine	13.73	C07187	277.2042
7	Sitostenone	13.71		412.3705
8	25-Hydroxyvitamin D2-25-glucuronide;25-Hydroxyvitamin D2 25-(beta-glucuronide)	13.7	C03033	588.3662
9	Allocryptopine	13.59	C02134	369.1576
10	Astaxanthin	13.56	C08580	596.3866
11	Mulberrofuran Q	13.28		592.1370
12	Broussonin C	13.23	C09524	312.1725
13	1-Isomangostin	13.16	C10071	410.1729
14	Lutein	13.14	C08601	568.4280
15	8,9-DiHETrE	13.03	C14773	338.2457
16	Tetrodotoxin	12.98	C11692	319.1016
17	Deguelin	12.92	C10417	394.1416
18	Palmitic acid	12.92	C00249	256.2402
19	Palmitoylethanolamide	12.61	C16512	299.2824
20	Abyssinone V	12.6	C09319	408.1937
21	7-(4-Hydroxyphenyl)-1-phenyl-4-hepten-3-one	12.53		280.1463
22	Betulin	12.32	C08618	442.3811
23	3-O-Acetyl-11-keto-beta-boswellic acid	12.3		512.3502
24	Levamisole	12.04	C07070	204.0721
25	Shionone	12	C17966	426.3862
26	Methyl linoleate	12		294.2559
27	Cinchonine	11.99	C06528	294.1732
28	(-)-Cinchonidine	11.99	C11379	294.1732
29	6-Gingerol; 6-Gingerol;[6]-Gingerol;Gingerol	11.99	C10462	294.1831
30	9-OxoODE	11.98	C14766	294.2195
31	13-HPODE;13-L-Hydroperoxylinoleic acid	11.98	C04717	312.2301
32	Colneleate	11.97	C19827	294.2195
33	(10E,12Z)-(9S)-9-Hydroperoxyoctadeca-10,12-dienoic acid	11.96	C14827	312.2301
34	N1-(alpha-D-ribosyl)-5,6-dimethyl-benzimidazole	11.88	C05775	278.1267
35	Cannabinol	11.75	C07580	310.1933
36	Sterebin A	11.75		310.2144
37	Karanjin	11.67		292.0736

38	Lansiumarin A	11.65		352.1311
39	p-Cymene	11.62	C06575	134.1096
40	Exemestane	11.61	C08162	296.1776
41	Progesterone	11.6	C00410	314.2246
42	Bisabolol oxide A	11.5	C16773	238.1933
43	13(S)-HPOT	11.47	C04785	310.2144
44	Ginkgolic acid C17:1	11.46		374.2821
45	(13E)-11a-Hydroxy-9,15-dioxoprost-13-enoic acid	11.44	C04654	352.2250
46	Methyl tanshinonate	11.43		338.1154
47	Levonorgestrel	11.29	C08149	312.2089
48	Valerenic acid	11.24	C09743	234.1620
49	Biochanin A	11.21	C00814	284.0685
50	1-Phenylethanol	10.56	C07112	122.0732
51	Geranylacetone	10.51	C13297	330.2923
52	Liquiritigenin	9.65	C09762	256.0736
53	Anacrotine	9.55	C10277	351.1682
54	Picrocrocin	9.25	C17055	330.1679
55	Diosmetin	9.15	C10038	300.0634
56	Macamide B	8.85		345.3032
57	Pinobanksin	8.14	C09826	272.0685
58	3-(2,3-Dihydroxyphenyl)propanoate	7.99	C04044	182.0579
59	Fulvine	7.99	C10304	309.1576
60	(-)-Sativan	7.89	C10526	286.1205
61	Herniarin	7.69	C09268	176.0473
62	Chrysophanol;Chrysophanic acid; Chrysophanol	7.46	C10315	254.0579
63	2-Hydroxyxanthone	7.27		212.0473
64	Peonidin-3-glucoside	6.93	C12141	462.1162
65	Bovinic acid	6.86	C04056	280.2402
66	Indole-3-carboxaldehyde	6.73	C08493	145.0528
67	Lumichrome	6.7	C01727	242.0804
68	Diosmin	6.59	C10039	608.1741
69	Rhoifolin	6.5	C12627	578.1636
70	D-Arabinose 5-phosphate	6.29	C01112	230.0192
71	Okanin	5.99	C08724	288.0634
72	Vanillin	5.9	C00755	152.0473
73	Eurycomalactone;Ingenol	5.79	C08759;C09112	348.1573
74	3-Ethoxy-4-hydroxybenzaldehyde;Ethyl salicylate	5.72		166.0630
75	indolin-2-one	5.67	C12312	133.0528
76	3-Indoleacetonitrile	5.46	C02938	156.0687
77	Terpinine-4-ol	5.45	C17073	154.1358
78	2-Hydroxycinnamic acid	5.44	C01772	164.0473
79	3-Methoxy-4,5-methylenedioxycinnamaldehyde	5.04		206.0579
80	4-Sulfobenzoate	4.68	C02236	201.9936

81	Caffeine	4.5	C07481	194.0804
82	Methyl gallate	4.44		184.0372
83	N-Feruloyl putrescine	4.33	C10497	264.1474
84	trans-Zeatin-riboside	4.31	C16431	351.1543
85	3,4-Dihydroxybenzaldehyde;Protocatechuic aldehyde; 3,4-Dihydroxybenzaldehyde	4.29	C16700	138.0317
86	Cinnamyl cinnamate	4.26		264.1150
87	Esculin	4.23	C09264	340.0794
88	Carbendazim	4.1	C10897	191.0695
89	Indole	3.82	C00463	117.0578
90	Deethylatrazine	3.81	C06559	187.0625
91	3-Methylindole	3.81	C08313	131.0735
92	Vasicine	3.76	C10733	188.0950
93	Syringic acid	3.57	C10833	198.0528
94	Coniferyl alcohol	3.27	C00590	180.0786
95	N-Nitroso-pyrrolidine	3.11	C19285	100.0637
96	Calystegine B2	2.95	C10851	175.0845
97	L-Phenylalanine;D-(+)-Phenylalanine;DL-Phenylalanine	2.82	C00079;C02265;C02057	165.0790
98	Phenethylamine	2.82	C05332	121.0891
99	5-(3-Pyridyl)-2-hydroxytetrahydrofuran	2.8	C19578	165.0790
100	5-Phenyl-1,3-oxazinane-2,4-dione	2.8	C16596	191.0582
101	4-Methyl-5-thiazoleethanol	2.66	C04294	143.0405
102	N1-Methyl-2-pyridone-5-carboxamide	2.63	C05842	152.0586
103	Adenosine	2.58	C00212	267.0968
104	2(3H)-Benzothiazolethione	2.57	C14437	166.9863
105	N,N-Dimethyl-1,4-phenylenediamine	2.56	C04203	136.1000
106	Vidarabine	2.28	C07195	267.0968
107	Guanosine 3',5'-cyclic monophosphate	2.26	C00942	345.0474
108	Adenosine 2',3'-cyclic phosphate	1.86	C02353	329.0525
109	Denudatine	1.59	C08680	343.2511
110	2-(Methylamino)benzoic acid	1.58	C03005	151.0633
111	7-Methylguanine	1.56	C02242	165.0651
112	Pyridoxine	1.55	C00314	169.0739
113	(2E)-Decenoyl-ACP;L-Pipecolic acid;Pipecolic acid	1.47	C03969;C00408	129.0790
114	Glycerophosphocholine	1.47	C00670	257.1028
115	cis-Aconitic acid	1.46	C00417	174.0164
116	3-Hydroxy-2-methylpyridine	1.44		109.0528
117	CYS-GLY;Cysteinylglycine	1.38	C01419	178.0412
118	2-Picolinic acid	1.33	C10164	123.0320
119	4-Guanidinobutyric acid	1.31	C01035	145.0851
120	4-Nitrocatechol	1.24	C02235	155.0219
121	3-Octyl alcohol	1.23	C17144	130.1358
122	Maltotriose	1.23	C01835	504.1690

123	Ethyl isovalerate	1.19	C12290	130.0994
124	5-Aminovaleric acid	1.11	C00431	117.0790
125	Nicotinamide	1.01	C00153	122.0480
126	2-Aminoisobutyric acid	0.98	C03665	103.0633
127	Maltol	0.9	C11918	126.0317
128	Sucrose	0.89	C00089	342.1162
129	Trigonelline	0.82	C01004	137.0477
130	Turanose	0.79	C19636	342.1162
131	Lactulose	0.77	C07064	342.1162
132	D-Proline	0.76	C00763	115.0633
133	dUMP	0.76	C00365	308.0410
134	D-Aspartic acid	0.76	C00402	133.0375
135	5-Methyl-2-furaldehyde	0.76	C11115	110.0368
136	alpha-D-Glucose;D-Tagatose	0.76	C00267;C00795	180.0634
137	2-Hydroxyethanesulfonate	0.76	C05123	125.9987
138	Beta-D-Fructose 2-phosphate	0.75	C03267	260.0297
139	Gemcitabine	0.75	C07650	263.0718
140	Proline betaine	0.74	C10172	143.0946
141	1-Kestose	0.74	C03661	504.1690
142	Ethyl caproate	0.74		144.1150
143	Proline;L-Proline	0.73	C00148	115.0633
144	Nicotinic acid	0.73	C00253	123.0320
145	Stachyose	0.73	C01613	666.2219
146	Streptozotocin	0.72	C07313	265.0910
147	Kojibiose	0.72	C19632	342.1162
148	Methylimidazole acetaldehyde	0.71	C05827	124.0637
149	Cytidine	0.7	C00475	243.0855
150	L-Acetylcarnitine	0.69	C02571	203.1158
151	L-Pipecolic acid	0.69	C00408	129.0790
152	Pregabalin	0.69		159.1259
153	O-Acetylethanolamine	0.67		103.0633
154	Phosphorylcholine	0.67	C00588	183.0660
155	L-Homoserine	0.67	C00263	119.0582
156	Pyrrolidonecarboxylic acid	0.67	C02237	129.0426
157	4-Aminobutyric acid	0.66	C00334	103.0633
158	L-Citruline;L-Citrulline	0.66	C00327	175.0957
159	L-Glutamic acid	0.66	C00025	147.0532
160	D-Serine	0.66	C00740	105.0426
161	D-Glutamine	0.66	C00819	146.0691
162	(S)-2-Aceto-2-hydroxybutanoate	0.66	C06006	146.0579
163	Sulforaphane	0.66		177.0282
164	Aminomalonic acid	0.65	C00872	119.0219
165	D-alpha-Aminobutyric acid	0.65	C02261	103.0633

166	L-Threonine	0.64	C00188	119.0582
167	L-Alanine;DL-Alanine	0.64	C00041;C01401	89.0477
168	L-Lysine;L-Glutamine	0.64	C00047;C00064	146.1055
169	2-Hydroxybutanoic acid	0.64	C05984	104.0473
170	Maltotetraose	0.64	C02052	666.2219
171	4-Hydroxybutanoic acid	0.64	C00989	104.0473
172	L-Aspartic acid	0.63	C00049	133.0375
173	3-Methy-L-histidine	0.61	C01152	169.0851
174	L-Arginine	0.6	C00062	174.1117
175	5-oxoproline	0.56	C01879	129.0426
176	Pimelic acid	0.55	C02656	160.0736
177	Ergothioneine	0.52	C05570	229.0885

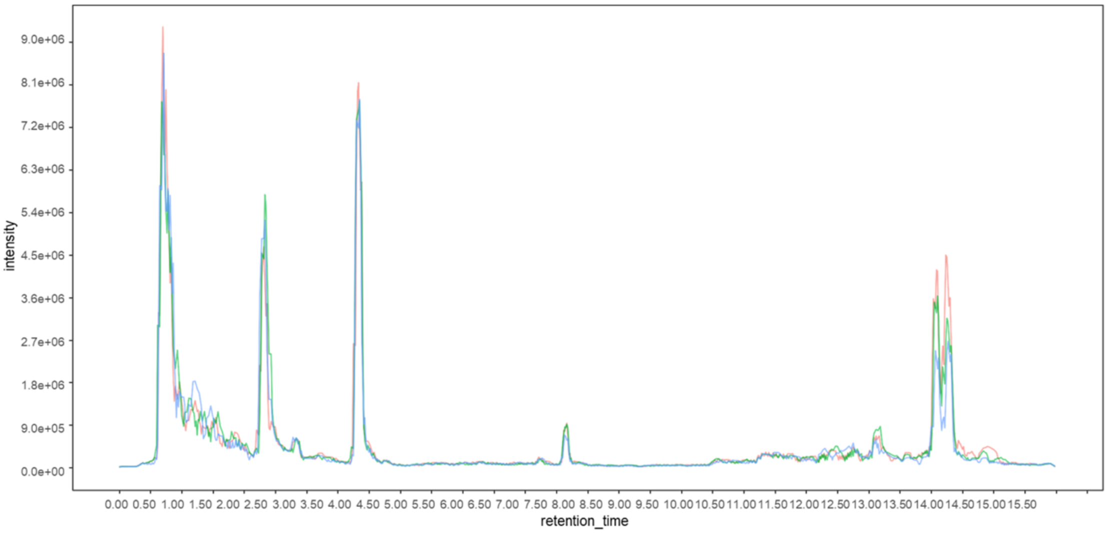

Figure S1. Overlaid total ion current chromatograms (TICs) of all QC samples.

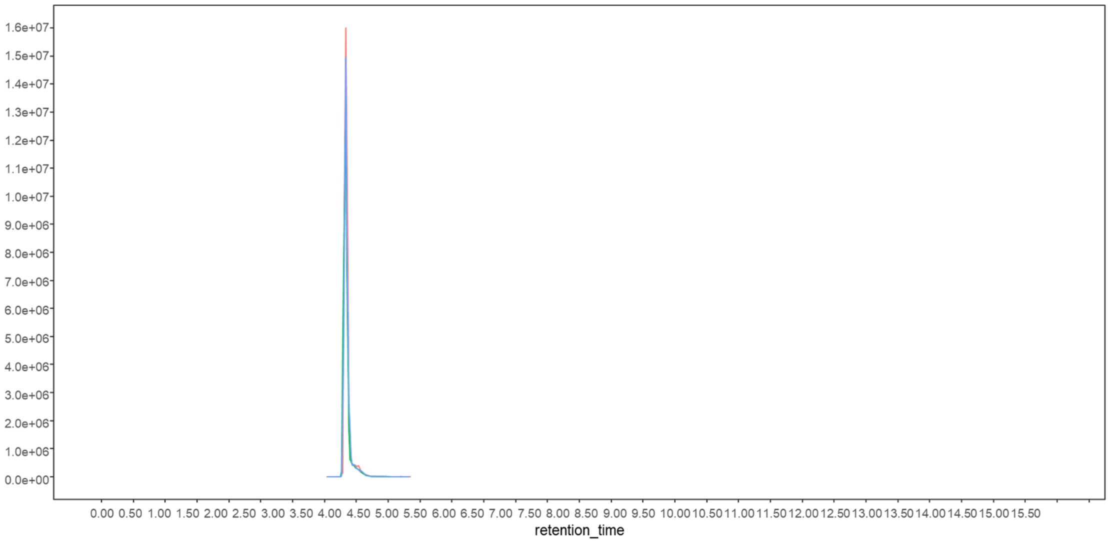

Figure S2. Overlaid extracted ion current chromatograms (EICs) of internal standard 2-chlorophenylalanine in QC samples.
